# High-Accuracy Relative Biological Effectiveness Values Following Low-Dose Thermal Neutron Exposures Support Bimodal Quality Factor Response with Neutron Energy

**DOI:** 10.3390/ijms23020878

**Published:** 2022-01-14

**Authors:** Laura C. Paterson, Amy Festarini, Marilyne Stuart, Fawaz Ali, Christie Costello, Chad Boyer, Ronald Rogge, Norma Ybarra, John Kildea, Richard B. Richardson

**Affiliations:** 1 Canadian Nuclear Laboratories (CNL), Chalk River, ON K0J 1J0, Canada; laura.paterson@cnl.ca (L.C.P.); amy.festarini@cnl.ca (A.F.); marilyne.stuart@cnl.ca (M.S.); fawaz.ali@cnl.ca (F.A.); christie.costello@cnl.ca (C.C.); chad.boyer@cnl.ca (C.B.); ron.rogge@cnl.ca (R.R.); 2 Division of Experimental Medicine, McGill University, Montreal, QC H4A 3J1, Canada; norma.ybarra@mcgill.ca; 3 Medical Physics Unit, Montreal, QC H4A 3J1, Canada; john.kildea@mcgill.ca

**Keywords:** RBE, thermal neutron, dicentric chromosome, DCA, micronucleus, CBMN, biological dosimetry

## Abstract

Theoretical evaluations indicate the radiation weighting factor for thermal neutrons differs from the current International Commission on Radiological Protection (ICRP) recommended value of 2.5, which has radiation protection implications for high-energy radiotherapy, inside spacecraft, on the lunar or Martian surface, and in nuclear reactor workplaces. We examined the relative biological effectiveness (RBE) of DNA damage generated by thermal neutrons compared to gamma radiation. Whole blood was irradiated by 64 meV thermal neutrons from the National Research Universal reactor. DNA damage and erroneous DNA double-strand break repair was evaluated by dicentric chromosome assay (DCA) and cytokinesis-block micronucleus (CBMN) assay with low doses ranging 6–85 mGy. Linear dose responses were observed. Significant DNA aberration clustering was found indicative of high ionizing density radiation. When the dose contribution of both the ^14^N(n,p)^14^C and ^1^H(n,γ)^2^H capture reactions were considered, the DCA and the CBMN assays generated similar maximum RBE values of 11.3 ± 1.6 and 9.0 ± 1.1, respectively. Consequently, thermal neutron RBE is approximately four times higher than the current ICRP radiation weighting factor value of 2.5. This lends support to bimodal peaks in the quality factor for RBE neutron energy response, underlining the importance of radiological protection against thermal neutron exposures.

## 1. Introduction

**Figure 1:**
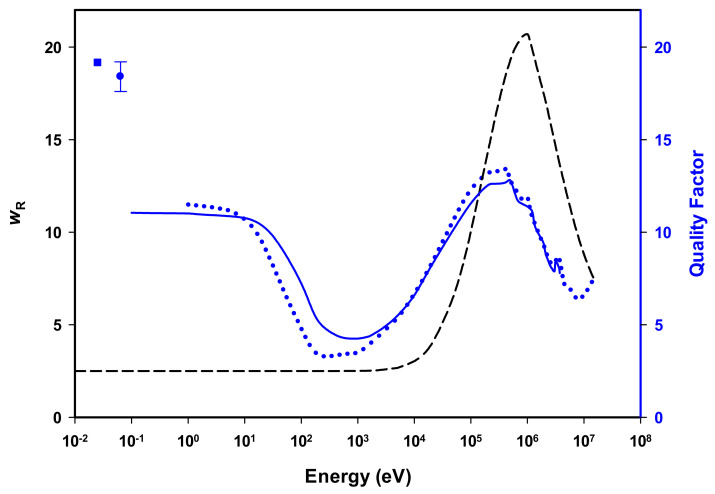
Illustration showing comparison of ICRP neutron radiation weighting factor $w_{R}$ (black dashed line) [10], and smoothed quality factor distributions calculated by Cross and Ing (blue dotted line) [13] and by Stinchcomb and Borak (blue solid line) [14], and thermal neutron quality factors calculated by Schuhmacher and Seibert (blue square) [11] and Ali et al. (blue circle) [12].

Secondary neutrons generated during proton and high-energy photon radiotherapy have been highlighted as a source of additional patient dose [1] and a risk factor for second malignant neoplasms [2,3]. Low-energy thermal neutrons comprise a notable fraction of these radiotherapy neutron fields [1]. Thermal neutrons have also been measured in CANada Deuterium Uranium (CANDU) reactor workplaces [4], in space [5,6], in high-altitude aircraft [7], and on the lunar surface [8]. Approximately 50% of thermal neutrons penetrate up to 2 cm into a tissue phantom, with a maximum range of over 10 cm [9]. The current recommendations from the International Commission on Radiological Protection (ICRP) indicate a radiation weighting factor ($w_{R}$) for thermal neutrons of 2.5, in contrast to a weighting factor of unity for the sparse ionization of low linear energy transfer (LET) radiation such as photons and electrons [10]. Neutron $w_{R}$ is a continuous function with energy, increasing from 2.5 at thermal and epithermal energies, up to a maximum of 20 at 1 MeV. Other high-LET particles, such as alpha particles, fission fragments, and heavy nuclei have also been ascribed a $w_{R}$ of 20 [10]. Monte Carlo simulations have indicated that the quality factor for thermal neutrons may be closer to 20 [11,12], and previous first-principles calculations and microdosimetric evaluations have demonstrated that the energy-dependent neutron quality factor follows a bimodal distribution with a peak near the thermal-neutron energy range and another in the fast-neutron energy range [13,14] (Figure 1). To investigate the discrepancy between the ICRP radiation weighting factor, $w_{R}$ and the reported quality factors, we experimentally evaluated the relative biological effectiveness (RBE) of low-dose, low-energy thermal neutrons in human peripheral blood lymphocytes. The RBE is a dimensionless quantity that describes the ability of a particular radiation type to produce a certain biological effect when compared to a reference radiation that is typically low-LET gamma rays or X-rays. RBE from animal studies, cell-killing, and chromosome aberration analysis influence the theoretically derived ICRP $w_{R}$ values; however, unlike RBE, $w_{R}$ accounts for the varying biological effects induced by different radiation types and take into account all possible biological consequences of a particular radiation [10]. In contrast, RBE values vary with multiple parameters including dose, dose rate, biological endpoint, cell type, cell-cycle phase, sample volume, sample depth, and microenvironment [15]. As a result, RBE and $w_{R}$ values are similar but not necessarily equal.

Evaluations of the dicentric chromosome assay (DCA) RBE_M_ of low-energy thermal neutrons in human peripheral blood lymphocytes are reported in several previous publications (Table 1), with maximum RBE (RBE_M_) results ranging from 10.8 ± 1.8 to 51.1 ± 31.3 [16,17,18,19]. The wide range of DCA RBE values and the large associated errors makes pinpointing a single thermal neutron RBE value difficult. There are currently no studies describing thermal neutron RBE using alternative endpoints. The International Atomic Energy Agency recommends cytogenetic assays used for dose assessment for photon equivalent dose range 0.1–5 Gy for the DCA and 0.3–4 Gy for the cytokinesis-block micronucleus assay (CBMN) assay [20]. However, the high ionization density and greater DNA damage potential of the thermal neutron exposures justify use of these assays at lower absorbed doses.

Neutron irradiation can cause elevated levels of complex DNA lesions, notably double-strand break (DSB) clusters and non-DSB clusters, compared to X-ray radiation [25].

Experimental evaluations have demonstrated that high ionization density exposure results in clustering of DNA double strand breaks but similar DNA repair kinetics as compared to gamma [26]. Previous thermal neutron DCA studies cover evaluations of the presence of aberration clustering, leading to the predicted non-Poisson over-dispersion of dicentric and ring chromosomes at doses above 300 mGy [18,19]. Complex DNA DSB lesions, such as those generated by neutron exposure, are more difficult to repair than the lesions generated by low-LET radiation, and may lead to genomic instability or carcinogenesis.

To facilitate thermal neutron RBE research, modifications were made to a neutron-scattering beam line at the National Research Universal (NRU) reactor of the Canadian Nuclear Laboratories (CNL) to allow for biological sample exposures [12]. Two assays were used to quantify the effects of low-dose thermal neutrons: the DCA and the CBMN assay. The DCA is currently the gold-standard method used to assess absorbed dose following accidental radiation overexposures or large-scale radiological events [20]. This method evaluates DNA DSB mis-repair in G_0_ lymphocytes. In previous studies, a correlation has been demonstrated between the yield of radiation-induced dicentric and ring chromosomes and absorbed dose for in vivo and in vitro exposures for low- and high-LET radiations [20]. This assay can also be used to evaluate DNA DSB aberration clustering by testing for compliance with the Poisson distribution. The CBMN assay, which also evaluates DNA DSB-related events in G_0_ lymphocytes, has been proposed as a higher-throughput alternative to the DCA, as micronuclei induction likewise scales well with radiation dose. However, unlike the DCA, the background rate of micronucleus (MN) generation varies significantly among individuals. Factors such as age, gender, diet, and smoking status have been found to impact endogenous MN frequency [27,28]. This work follows from a previous experimental low-dose neutron RBE investigation at CNL using ^252^Cf fast neutrons [29].

The original aim of this study was twofold: to investigate DNA aberration clustering in the low-dose range, and to experimentally evaluate high-accuracy RBE for thermal neutrons. In view of the unexpectedly high RBE value acquired, a secondary aim was to provide some validity to the findings, as the high RBE value is commensurate with theoretical quality factor analyses for thermal neutrons carried out by microdosimetry and first-principles calculations.

## 2. Results

### 2.1. Dicentric Chromosome Assay

The DCA aberration distribution for three donors following 64 meV thermal neutron exposure is presented in Table 2. A total of 13,819 cells from three donors were scored across eight dose points, up to a total dose of 82.1 mGy, yielding 273 dicentric and ring chromosome aberrations. The background yield was 0.0004 aberrations per cell, and the maximum aberration yield was 0.0627 aberrations per cell. The majority of cells had no aberrations. At the highest dose point of 82.1 mGy, only approximately 6% of cells contained between one and three dicentric and/or centric ring aberrations. Five of the seven irradiated data points demonstrated non-Poisson over-dispersion with values of *u* > 1.96 (Table 2). Only the 12.0 mGy and 23.9 mGy dose points did not achieve the predicted over-dispersion.

**Figure 2:**
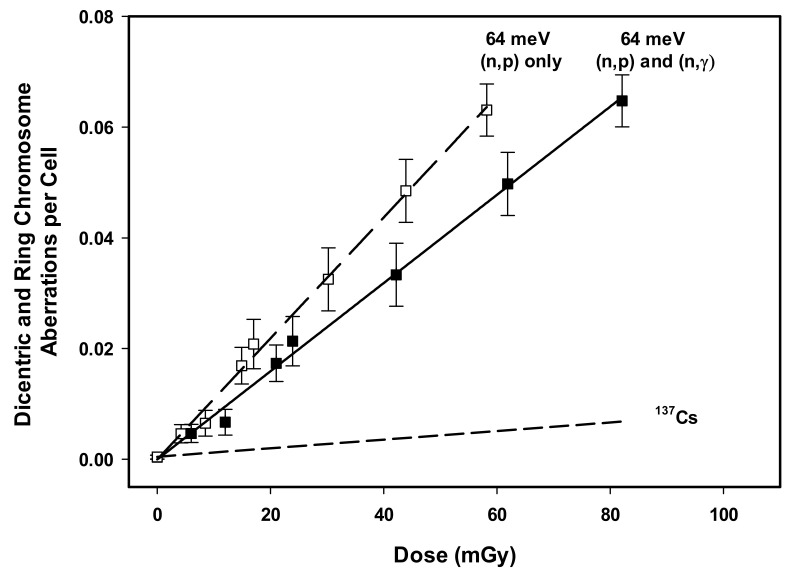
DCA linear dose response regression for the 64 meV neutron exposures ((n,p) and (n,γ), filled squares and solid line; only the (n,p) reaction, open squares and long-dash line), compared to the extrapolated ^137^Cs curve (short-dash line) from the linear-quadratic dose response derived by Flegal et al. [21]. The graph details the relationship between radiation dose and the number of dicentric and centric ring chromosome aberrations per cell. Error bars represent standard error of the mean across three donors.

Figure 2 illustrates the linear 64 meV thermal neutron dose response curves compared to the extrapolated ^137^Cs reference radiation dose response curve [21]. Data points represent the mean of three independent experiments involving blood from three different donors. Error bars illustrate the standard error of the mean. Linear and linear-quadratic regressions were both evaluated, revealing a preference for a linear dose response due to the non-significant *z*-test result for the *β* regression coefficient. The regression coefficients, the associated standard error values, results of the *χ*^2^- and *z*-tests, and the *R*^2^ values are presented in Table 3.

### 2.2. Cytokinesis-Block Micronucleus Assay

**Figure 3:**
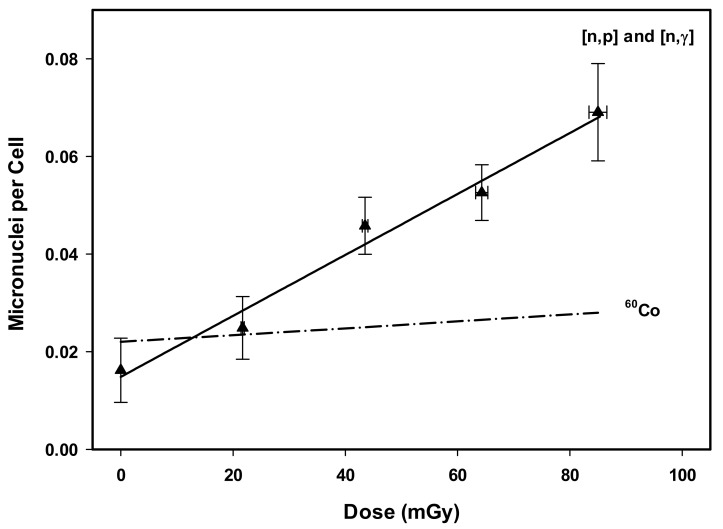
CBMN linear dose response regression (filled triangles, solid line) for 64 meV thermal neutron exposures compared to the extrapolated ^60^Co curve (dash-dot line) generated from the linear quadratic dose response derived by McNamee et al. [30], detailing the relationship between radiation dose and the number of micronuclei per cell. Error bars represent standard error of the mean across three donors.

Employing the CBMN assay, a total of 75,000 bi-nucleated (BN) cells from three independent donors were examined across five dose points, yielding 3182 micronuclei. As a function of dose, the aberration yield following exposure to 64 meV neutrons varied between 243 aberrations in 15,000 BN cells at 0 mGy to 1036 aberrations in 15,000 BN cells at 85.0 ± 1.6 mGy (Table 4). Therefore, the background yield was 0.016 MN per cell, and the maximum aberration yield was 0.069 MN per cell. The majority of cells had no MN, even at the highest dose point where approximately 7% of cells revealed one to three MN. Highly damaged cells with three MN represent only 0.07% at the highest dose. The average proliferative index for the 0 mGy control sample was found to be 1.7. Standard errors for dose and MN frequency represent the difference across the three blood donors. Figure 3 illustrates the linear 64 meV thermal neutron CBMN dose response curve and the extrapolated ^60^Co reference radiation dose response curve [30]. All five exposures demonstrated non-Poisson over-dispersion with values of *u* > 1.96, including the 0 mGy dose point, which is a characteristic of the CBMN assay (Table 4). Linear and linear-quadratic regression were both evaluated. As with the DCA, there was a preference for linear regression due to the non-significant *z*-test result for the *β* coefficient. The regression coefficients, the associated standard error values, results of the *χ*^2^- and *z*-tests, and the *R*^2^ values are presented in Table 5.

### 2.3. Relative Biological Effectiveness

For the DCA in which the nitrogen, ^14^N(n,p)^14^C and hydrogen, ^1^H(n,γ)^2^H capture reactions were considered together, a DCA RBE_M_ value of 11.3 ± 1.6 was found when referenced to ^137^Cs exposures [21]. Similarly, when the results of only the ^14^N(n,p)^14^C reaction was evaluated, an elevated, but not significantly different, RBE_M_ of 15.5 ± 2.2 was revealed (*z* = 1.54, *p* = 0.123). As presented in Table 2, dose-specific DCA RBE values decreased with increasing dose and varied from 11 at 6 mGy to 7 at 82.1 mGy, when both the ^14^N(n,p)^14^C and ^1^H(n,γ)^2^H capture reactions were considered, and from 15 to 10 when the effect of only the ^14^N(n,p)^14^C capture reaction was evaluated.

The CBMN assay revealed an RBE_M_ value of 9.0 ± 1.1 for both the ^14^N(n,p)^14^C and ^1^H(n,γ)^2^H capture reactions when referenced to ^60^Co radiation [30]. Dose-specific CBMN RBE values decreased with increasing dose, revealing an RBE of 8 at the lowest dose of 21.7 mGy and an RBE of 7 at the highest dose of 85.0 mGy.

## 3. Discussion

### 3.1. DNA Aberration Clustering

The majority of DCA dose points exhibited non-Poisson over-dispersion (Table 2). This confirms the presence of DNA aberration clustering, and is consistent with results from the higher-dose thermal neutron DCA studies [18,19]. Therefore, thermal neutron exposures in lymphocytes exhibit the track structure characteristics and associated DNA damage of high-LET radiations, which does not match the current ICRP $w_{R}\;$ recommendation of 2.5. In biological dosimetry, traditionally 50 or fewer DCA cells are scored for triage biological dosimetry, and 500 to 1000 DCA cells are scored for detailed dose estimates [20,31]. Although over-dispersion of chromosome aberrations is considered a hallmark of high-LET radiation exposure, the data in Table 2 demonstrate that over-dispersion may not be immediately evident in low-dose neutron samples. For example, for the dose points that achieved over-dispersion, the removal of up to four cells containing two or more dicentric or ring chromosome aberrations per dose point was enough to rescind the over-dispersion effect in a sample size of over 1400. The failure to observe the predicted high-LET over-dispersion is especially likely for triage biological dosimetry where lower numbers of cells are scored; however, as demonstrated by the current study, over-dispersion is also possible when a higher numbers of cells are evaluated.

All CBMN assay data points were found to exhibit non-Poisson over-dispersion (Table 4). This finding was expected, as the tendency for over-dispersion is well documented for the CBMN assay following both low-LET photon exposures [20] and high-LET fast-neutron exposures [32]. This is theorized to be due to the large number of cells with zero aberrations, a positive concurrence between micronuclei, or an association between the radiation dose and the associated error [33].

### 3.2. Bimodal Neutron RBE Values

Using the *z*-test with Holm’s method to evaluate multiple comparisons, there was insufficient evidence to conclude that our DCA RBE_M_ value of 11.3 ± 1.6 (for both ^1^H(n,γ)^2^H and ^14^N(n,p)^14^C reactions) was different from any of the previously published RBE_M_ data for thermal neutrons, including the values of 10.8 ± 1.8 found using data from Sevan’kaev et al. [16] (calculated by Schmid et al. [18]), 36.4 ± 13.3 reported by Schmid et al. [18], and 51.1 ± 31.3 described by Sasaki et al. [19]. This seemingly surprising result is partially due to the high variance of the latter two studies. For Schmid et al. [18] and Sasaki et al. [19], which revealed the largest RBE values of 36.4 ± 13.3 and 51.1 ± 31.3, respectively, the very low *α* regression coefficient of the gamma reference radiation (Table 1) played a significant role in the elevated RBE values.

Our data suggest that the linear dose response function is preferred for all endpoints. This finding is consistent with those of previous thermal neutron DCA studies [16,17,18]. As shown in Table 1, many of the previous thermal neutron DCA studies reported large RBE standard error values, or reported no error estimate at all.

There was no significant difference between our DCA RBE_M_ of 11.3 ± 1.6 and the CBMN RBE_M_ of 9.0 ± 1.1 (*z* = 1.13, *p* = 0.250), for both ^1^H(n,γ)^2^H and ^14^N(n,p)^14^C reactions. As both assays examine chromosome aberrations initiated by DNA DSBs, it is not surprising that the RBE_M_ is similar.

During the irradiation period, a very low-dose gamma field was generated at the site of the thermal neutron exposures as consequence of gamma radiation escaping into the reactor beamline. This gamma field was not quantified, but control sample evaluations demonstrated that the total dose delivered to the samples was below the DCA threshold of gamma-ray detection of 0.1 Gy [21]. Previous studies have demonstrated that very low doses of gamma radiation in conjunction with, or in advance of, fast-neutron exposure can result in a protective effect whereby DNA damage is reduced in mixed-radiation exposures of peripheral blood lymphocytes [34,35]. It has been proposed that a hormetic effect caused by these low-dose gamma fields may be responsible for the large variation of RBE values found in the literature [35]. Although there is notable DCA RBE variation across thermal neutron studies (Table 1), external photon fields generated as a consequence of the neutron irradiation setup have not been identified in other publications. As a result, it is not possible to comment on whether radiation hormesis played a role in the large range of reported, but not statistically different, thermal neutron RBE values.

Absorbed dose in the current test system is a result of two neutron capture reactions. The ^14^N(n,p)^14^C reaction, accounts for approximately 71% of the total absorbed dose to blood in rotating quartz tubes. Tertiary electrons from the ^1^H(n,γ)^2^H capture reaction deliver the remaining absorbed dose (29%) [12]. When only the ^14^N(n,p)^14^C reaction was considered, an elevated DCA RBE_M_ value of 15.5 ± 2.2 was revealed (Table 1). This value approaches the theoretical quality factor of 19.17 calculated by Schuhmacher and Siebert [11] for the thermal neutron ^14^N(n,p)^14^C capture reaction. This RBE_M_ value of 15.5 ± 2.2 is also similar to the quality factor of 18.4 ± 0.8 simulated by Ali et al. [12] for the current experimental setup. Furthermore, all RBE_M_ values presented here support the bimodal quality factor distribution put forward by Cross and Ing [13] and Stinchcomb and Borak [14] that describes one peak in quality factor within, or nearing, the thermal range and a second peak in the fast-neutron energy range (Figure 1). Cross and Ing’s quality factor results were obtained by first-principles calculations for tissue [13], whereas the data trend of Stinchcomb and Borak was acquired using microdosimetry [14].

**Figure 4:**
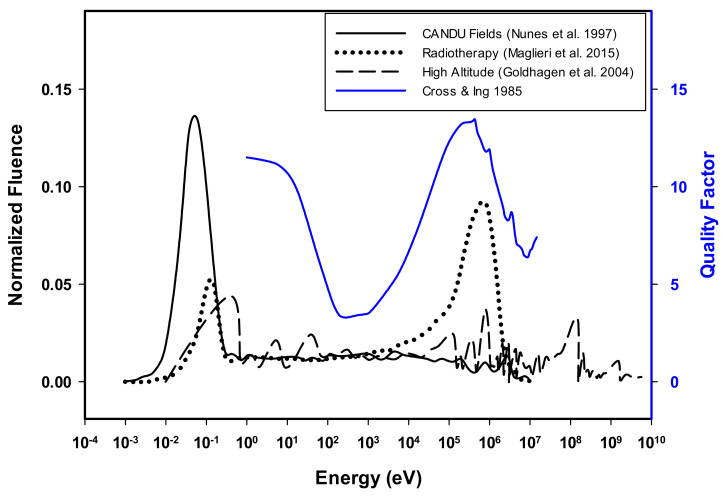
Illustration showing comparison between the smoothed normalized fluence peaks present in radiotherapy treatment facilities [1], CANDU reactor environments [4], and at high altitudes [7] (originally normalized and presented by Ali et al. [12]) and the bimodal quality factor distribution presented by Cross and Ing [13].

It is acknowledged that RBE is expected to fall in value for targets deeper into the body. Ali et al. [12] calculated 23% and 19% of the total absorbed dose for ^14^N(n,p)^14^C reactions in the International Commission on Radiation Units and Measurements sphere at the eye lens’ depth of 3 mm and deep organs’ depth of 10 mm, respectively, Notwithstanding, the thermal neutron experimental RBE data presented here support bimodal peaks for RBE versus neutron energy, which complements the theoretical quality factor data. This finding may have a significant influence on radiation protection guidelines because the bimodal peaks in quality factor nearly overlay the fluence peaks measured in CANDU reactor workplaces [4], in radiotherapy treatment bunkers [1], and at high altitudes [7], as presented in Figure 4, indicating a high potential for DNA damage for the neutrons encountered in these environments. Consequently, knowledge and understanding of this bimodal trend of the neutron quality factor and, subsequently, of the RBE means that radiation protection aspects that specifically acknowledge a thermal neutron peak warrant further consideration.

### 3.3. RBE Accuracy

There was a gamma dose rate discrepancy between the 64 meV thermal neutrons exposures and the gamma reference radiation for each endpoint. All thermal neutron exposures had an average dose rate of 22 mGy h^−1^. In contrast, the DCA ^137^Cs reference radiation exposures were performed using a dose rate of 49.8 Gy h^−^^1^ [21], and the CBMN reference radiation exposures were performed using two ^60^Co irradiators with dose rates of 11.2 Gy h^−1^ (for doses up 0.5 Gy) and 529.8 Gy h^−1^ (for doses between 1 Gy and 4 Gy). It is well-established that low-LET radiations are susceptible to dose rate effects [20], and as such it was not practicable to match the gamma dose rate with the very low thermal neutron dose rate of 22 mGy h^−1^. However, high-LET radiation, such as the thermal neutron exposures documented here, are not vulnerable to the same dose rate effects because the two lesions required for a dicentric or ring chromosome can be produced by a single track of radiation [20]. This has been previously demonstrated experimentally in human lymphocytes [36]. Other notable neutron RBE studies have accepted a mismatch between the neutron and photon dose rates [37,38,39]. Given this information, RBE calculations presented here, which use a thermal neutron dose rate of 22 mGy h^−^^1^ and a much higher photon dose rate, were deemed acceptable.

Publications regarding neutron RBE in lymphocytes are often related to biological dosimetry capability. To allow for comparison, whole blood irradiation and consequent lymphocyte phytohemagglutinin stimulation were chosen to allow for direct comparison to previous work. However, lymphocytes are known to be a more radiosensitive cell type [40], and care should be taken before extrapolating these results to other cell types.

This is the second peer-reviewed publication from our laboratory that evaluates the RBE of neutrons. An earlier study of ^252^Cf fast neutrons of 2.1 MeV (average energy) described DCA RBE_M_ values of 15.0 ± 2.2 and 25.7 ± 3.8 for the neutron-plus-gamma and neutron-only dose components, respectively [29]. Our thermal neutron DCA RBE_M_ values of 11.3 ± 1.6 (for both ^14^N(n,p)^14^C and ^1^H(n,γ)^2^H reactions) and 15.5 ± 2.2 (for ^14^N(n,p)^14^C reaction alone) are slightly lower than the ^252^Cf values, a finding that is consistent with those of other studies. Specifically, DCA RBE_M_ in human peripheral blood lymphocytes has been found to increase from slow-neutron energies (a few hundred eV) up to approximately 0.385 MeV (RBE_M_ = 94.4 ± 38.9) [41]. Beyond this point, the DCA RBE_M_ begins to decrease with increasing neutron energy, but does not reach unity. This trend corresponds with the currently ICRP recommended monomodal peak for quality factor that is approximately 20 nearing 1 MeV [10].

### 3.4. Limitations

The scoring of both the DCA and CBMN assay was done manually. To achieve high accuracy and precision, scoring was performed by a qualified individual within the Canadian biodosimetry network who undergoes annual network intercomparisons to ensure effective analysis methods [42]. To avoid scoring bias, samples were blinded prior to microscopy.

To generate dose response curves, data was pooled from three donors. Except for the 12.0 mGy dose point, 500 or more cells per donor per dose point were compiled for the DCA. At low cell numbers, it is possible that inter-individual differences in spontaneous dicentric frequency may have been missed.

For the RBE calculation at the low doses examined here, it was necessary to extrapolate the gamma dose response from the DCA and CBMN lines of best fit, rather than from matched data points. This is an unavoidable consequence of this low-dose work, and highlights the importance of research into novel biodosimetry assays that can quantify low-dose exposures.

It can be difficult to extrapolate RBE from in vitro blood exposures to whole-body exposure situations. The dose contribution of the ^1^H(n,γ)^2^H reaction is much lower in a test tube than for a whole body dose [10]. As mentioned in the section above on Bimodal Neutron RBE Values, the RBE values can be expected to be reduced for thermal neutron exposure of shallow targets such as the eyes and skin. Nevertheless, these results demonstrate that thermal neutrons can cause complex clustered damage in tissue and should not be dismissed as being less hazardous than higher energy neutrons.

## 4. Materials and Methods

### 4.1. Blood Donors

Blood samples were drawn by venipuncture from healthy blood donors at CNL (Chalk River, ON, Canada) into evacuated tubes containing sodium citrate anticoagulant (BD, Franklin Lakes, NJ, USA). All blood donors were volunteers who willingly agreed to participate in a research proposal for which the ethics had been approved by Veritas Independent Review Board (Montreal, QC, Canada). Donors were non-smokers who reported feeling healthy on the day of the blood draw and had no history of radiotherapy or chemotherapy treatment. Three donors, two males and one female, participated in the DCA and CBMN studies. Enrollment of three or fewer donors is common in neutron RBE studies [18,29,41,43,44]. Donors were between 20 and 50 years old. Following venipuncture, anticoagulated whole blood was immediately transferred into quartz test tubes and then transported to the irradiation facility.

### 4.2. Irradiation Conditions

The 64 meV thermal neutron irradiations were completed at CNL in the NRU reactor using the E3 spectrometer of the Canadian Neutron Beam Centre. The details of the beam-line configuration and the associated physical modelling of the test system were described previously by Ali et al. [12]. Briefly, a pyrolytic graphite (PG) crystal in the E3 spectrometer selected for an average neutron kinetic energy of 64 meV. The ^14^N mass fraction in blood was modelled at 2.96%. The total absorbed dose per unit neutron fluence delivered to a blood sample was calculated to be 0.274 pGy cm^2^ n^−1^ [12]. The ^14^N(n,p)^14^C capture reaction accounted for nearly 71% of the total absorbed dose, and tertiary electrons from the ^1^H(n,γ)^2^H capture reaction were responsible for the remaining absorbed dose. Quartz test tubes, each containing 4.2 mL of whole blood, were positioned on a computer-controlled gantry in front of the beam port. The tubes were rotated at 60 rpm during the irradiation period to ensure a uniform sample exposure [12] and were maintained at room temperature throughout the irradiation and transportation periods to minimize the effects of concurrent DNA repair [20]. The average neutron fluence rate was quantified using gold foil activation analysis. Due to frequent changes in the reactor power, the neutron fluence rate also varied with time. The average neutron fluence rate throughout the irradiation campaign was 2.25 × 10^7^ ± 0.03 × 10^7^ n cm^−2^ s^−1^ and the average absorbed dose rate delivered to the blood samples was 22 ± 0.9 mGy h^−1^. Cell cultures were established 18 h after exposure. This time interval ensured sufficient radionuclide decay for safe sample handling.

### 4.3. Assays

#### 4.3.1. Dicentric Chromosome Assay

Whole blood from three donors was irradiated with neutron doses of 6.0 mGy to 82.1 mGy. Duplicate cultures were established in Nunc T-25 flasks (Thermo Fisher Scientific Inc., Waltham, MA, USA). Samples were harvested for slide making and fluorescence-plus-Giemsa staining following a 48 h incubation according to the IAEA recommendations for biological dosimetry laboratories [20], as described previously by Paterson et al. [29]. Slides were blinded and metaphase spreads were imaged at 630× magnification using the Metafer slide scanning platform (Metasystems Group Inc., Newton, MA, USA). Complete metaphase spreads in first division were scored manually according to criteria described previously [29]. A minimum of 500 cells per donor per dose point were scored for all dose points, except for 12.0 mGy where only 1414 cells for the three donors combined were available (Table 2).

#### 4.3.2. Cytokinesis-Block Micronucleus Assay

Whole blood from three donors was exposed to 64 meV thermal neutrons. Doses ranged from 21.7 mGy to 85.0 mGy. Whole blood cultures were established according to a modified procedure of Fenech et al. [27], previously described by McNamee et al. [30]. Cultures were incubated for 72 h, with cytochalasin B (Millipore Sigma, Burlington, MA, USA) present for the final 28 h. Slides were blinded and stained with acridine orange to allow for fluorescence microscopy. Binucleated cells were manually scored at 400× magnification according to criteria provide by Fenech et al. [27]. A total of 5000 cells were scored per donor per dose point (Table 4).

### 4.4. Statistics

Statistical analysis for the DCA and CBMN assay was completed according to the IAEA cytogenetic dosimetry recommendations [20]. The results were tested for compliance with the Poisson distribution by calculating the dispersion index (variance, *σ*^2^/mean, *y*) and the *u*-test statistic. A dispersion index of unity confirms agreement with the Poisson distribution. Dispersion indices differing from unity were further tested using the *u*-test, where statistic values above 1.96 indicated non-Poisson over-dispersion at the 5% significance level [20,45].

The Dose Estimate software package (version 5.2) was used to ensure proper DCA and CBMN data curve-fitting [46]. Linear-quadratic regression is presented in the form *A* = *c* + *αD* + *βD*^2^, where *A* is the frequency of aberrations at a given dose point, *c* is the background frequency of aberrations, *α* is the linear dose response coefficient (aberrations produced by a single track of radiation), *β* is the quadratic dose response coefficient (aberrations produced by two tracks of radiation), and *D* represents the dose in Gy. Errors were reported as either standard deviation (SD) or standard error (SE). The chi-squared test (*χ*^2^) was used to evaluate the fit of the dose response curves, and the significance of the dose response equation coefficients was evaluated using the *z*-test. The *p*-values less than 0.05 were considered statistically significant.

### 4.5. Dose and RBE Calculations

The number of dicentric and ring chromosome aberrations attributable only to the (n,p) reaction were calculated using the method described by Schmid et al. [18]. Briefly, the dose contribution from the ^1^H(n,γ)^2^H capture reaction was calculated using the data for absorbed dose per unit neutron fluence previously derived by Ali et al. [12]. This information was used to calculate the number of photon-induced aberrations per cell at each dose point based on the previously published in-house ^137^Cs dose response curve [21]. The number of photon-induced aberrations per cell was then subtracted from the total number of aberrations per cell to give a value for the (n,p)-induced aberrations per cell. This method could not be applied to the CBMN assay data due to the differing background aberration rate between the 64 meV neutron samples and the reference ^60^Co dose response curve.

The RBE for 64 meV neutrons was calculated using two methods: the maximum RBE_M_ method, and the dose-specific RBE method. The RBE_M_ was calculated as the ratio of the regression equation *α*-coefficient values from the thermal neutron exposures to those of the photon dose response curves [20]. Dose-specific RBE values were calculated as the ratio of gamma dose to neutron dose required to produce the same effect.

## 5. Conclusions

A linear dose response relationship for thermal neutrons was found for DCA and CBMN assays following low doses from 64 meV thermal neutrons. High RBE_M_ values of 11.3 ± 1.6 and 15.5 ± 2.2 were found for the DCA, when all neutron capture reactions were considered and when only the ^14^N(n,p)^14^C reaction was considered, respectively. As expected, RBE_M_ of 9.0 ± 1.1 for the CBMN assay was similar to the DCA RBE_M_ when all neutron capture reactions were considered. These consistent, high-accuracy RBE values are about four times higher than the current ICRP $w_{R}$ value of 2.5 for thermal neutron exposures, but similar to the theoretical quality factors described for the thermal neutron ^14^N(n,p)^14^C capture reaction [11,12]. Our experimental RBE_M_ values lend support to a theoretically derived bimodal quality factor trend that describes not only a fast-neutron peak but also a second peak for elevated quality factor values in the low-energy range. This work accurately confirms that the low-energy thermal neutrons found in radiotherapy treatment bunkers, in CANDU reactor facilities, and during spaceflight can cause significant DNA damage in healthy tissues, which could theoretically result in genomic instability and mutation-induced carcinogenesis, cataracts, and other health effects. Future work should examine whether this phenomenon persists in cell types of differing radiosensitivity or in animal models. If substantiated, this bimodal trend in neutron quality factor justifies consideration in radiation protection recommendations.

## Figures and Tables

**Table 1:** Comparison of DCA RBE_M_ and linear regression (*α* coefficient) values in human peripheral blood lymphocytes following thermal neutron exposure.

Neutron Energy (meV)	Reference	Absorbed Dose Ranges (Gy)	# Dose Points	Regression		RBE_M_
				Neutron *α* ± SE (Gy-1)	Gamma *α* ± SE (Gy-1)	
64	This study	0.006–0.082	7	0.789 ± 0.045	0.070 ± 0.0088 ^a^	11.3 ± 1.6
Thermal	Sevan’kaev et al. [16]	0.16–0.64	3	0.745 ± 0.03 ^b^	0.069 ± 0.011 ^c^	10.8 ± 1.8 ^b^
Thermal	Wojcik et al. [17]		6	0.669 ± 0.002	0.055 ± 0.023	12.16
25.3	Schmid et al. [18]	0.375–1.875	5	0.400 ± 0.018	0.011 ± 0.004 ^d^	36.4 ± 13.3
25	Sasaki et al. [19]	0.073–2.19	7	0.920 ± 0.028	0.018 ± 0.011 ^e^	51.1 ± 31.3

The number of dose points does not include unirradiated control samples. ^a^ Flegal et al. [21]; ^b^ Recalculated by Schmid et al. [18]; ^c^ Recalculated by Lloyd & Edwards [22]; ^d^ Bauchinger et al. [23]; ^e^ Sasaki et al. [24].

**Table 2:** DCA aberration distribution in the peripheral blood lymphocytes of three donors and dose-specific RBE values following 64 meV thermal neutron exposure. Values of *u* demonstrating non-Poisson over-dispersion are highlighted in bold. The dose is reported as the mean dose with standard error (SE).

Total Dose ± SE (mGy)	(n,p) Dose (mGy)	(n,γ) Dose (mGy)	Cells Scored	Total Aberr. (±SD) *	Distribution of Aberr.				Total Aberr. per Cell	(n,p) Aberr. per Cell	(n,γ) Aberr. per Cell	*σ*^2^/*y*	*u*	(n,p) + (n,γ) RBE	(n,p) RBE
					0	1	2	3							
0 ± 0	0	0	2800	1 ± 1	2799	1	0	0	0.0004	0.0004	0	1.00	-.	-.	-
6.0 ± 0.2	4.2	1.8	1500	7 ± 3	1494	5	1	0	0.0047	0.0046	0.0001	1.28	8.34	11	15
12.0 ± 0.3	8.5	3.5	1414	11 ± 3	1404	11	0	0	0.0067	0.0065	0.0002	0.99	−0.20	7	10
21.0 ± 0.4	14.9	6.1	1500	26 ± 5	1478	18	4	0	0.0173	0.0169	0.0004	1.29	8.13	10	14
23.9 ± 0.6	16.9	7.0	1500	32 ± 6	1469	30	1	0	0.0213	0.0208	0.0005	1.04	1.16	10	14
42.2 ± 0.6	30.2	12.0	1500	50 ± 7	1456	39	4	1	0.0333	0.0325	0.0008	1.25	6.84	9	12
61.9 ± 0.7	43.8	18.1	2030	101 ± 10	1940	80	9	1	0.0498	0.0485	0.0013	1.19	6.03	8	11
82.1 ± 1.8	58.1	24.0	1575	102 ± 10	1487	75	12	1	0.0648	0.0631	0.0017	1.23	6.49	7	10

Aberr., Aberrations. * Assuming Poisson distribution.

**Table 3:** DCA linear dose response regression and *R*^2^ values for 64 meV thermal neutrons and ^137^Cs photons.

Radiation	Regression	*α* [±SE] (Gy^−1^)	*β* [±SE] (Gy^−2^)	*c* [±SE]	*χ*^2^-Test Sig.	*α z*-Test Sig.	Pearson’s *R*^2^ Value
64 meV (n,p) + (n,γ)	*A* = 0.0003 + 0.789*D*	0.789 ± 0.045	-	0.0003 ± 0.0017	0.9867	0.00001	0.998
64 meV (n,p) only	*A =* 0.0003 + 1.088*D*	1.088 ± 0.063	-	0.0003 ± 0.0021	0.9948	0.00001	0.999
^137^Cs [21]	*A* = 0.070*D* + 0.061*D*^2^	0.070 ± 0.0088	0.061 ± 0.0043	-	-	-	-

*A*, Aberrations per cell; *α*, *β*, *c*, regression coefficients; *D*, Dose (Gy); *R*^2^, coefficient of determination; Sig., Significance.

**Table 4:** CBMN distribution in the peripheral blood lymphocytes of three donors and dose-specific RBE values. Values of *u* demonstrating non-Poisson over-dispersion are highlighted in bold. Standard error of the mean represents the error for the dose across three donors.

Total Dose ± SE (mGy)	(n,p) Dose (mGy)	(n,γ) Dose (mGy)	Cells Scored	Total Aberr. (±SD *)	Distribution of Aberr.				Total Aberr. per Cell	*σ*^2^/*y*	*u*	(n,p) + (n,γ) RBE
					0	1	2	3				
0 ± 0	0	0	15000	243 ± 16	14757	216	12	1	0.0162	1.11	9.31	-
21.7 ± 0.3	15.4	6.3	15000	373 ± 19	14627	281	37	6	0.0249	1.27	23.42	8
43.5 ± 0.5	30.8	12.7	15000	687 ± 26	14313	499	82	8	0.0458	1.26	22.76	8
64.3 ± 1.1	45.5	18.8	15000	789 ± 28	14211	578	83	15	0.0526	1.27	23.53	7
85.0 ± 1.6	60.1	24.9	15000	1036 ± 32	13964	759	122	11	0.0691	1.23	19.89	7

Aberr., Aberrations. * Assuming Poisson distribution.

**Table 5:** CBMN linear dose response regression for 64 meV thermal neutrons and ^60^Co photons.

Radiation	Regression	*A* [±SE] (Gy^−1^)	*β* [±SE] (Gy^−2^)	*c* [±SE]	*χ*^2^-Test Sig.	*α z*-Test Sig.	Pearson’s *R*^2^ Value
64 meV (n,p) + (n,γ)	*A* = 0.0153 + 0.6152*D*	0.615 ± 0.052	-	0.0153 ± 0.0085	0.0013	0.0013	0.98
^60^Co [30]	*A* = 0.022 + 0.068*D* + 0.024*D*^2^	0.068 ± 0.006	0.024 ± 0.0020	0.022 ± 0.0020	-	-	-

Abbreviations: *A*: Aberrations per cell, *α*, *β*, *c*: regression coefficients, *D*: Dose (Gy), *R*^2^: coefficient of determination, Sig.: Significance.
